# Fumarase From *Cyanidioschyzon merolae* Stably Shows High Catalytic Activity for Fumarate Hydration Under High Temperature Conditions

**DOI:** 10.3389/fmicb.2020.560894

**Published:** 2020-09-16

**Authors:** Shoki Ito, Kaori Iwazumi, Haruna Sukigara, Takashi Osanai

**Affiliations:** School of Agriculture, Meiji University, Kawasaki, Japan

**Keywords:** fumarase, tricarboxylic acid cycle, l-malate, microalgae, cyanobacteria

## Abstract

Fumarases (Fums) catalyze the reversible reaction converting fumarate to l-malate. There are two kinds of Fums: Class І and ІІ. Thermostable Class ІІ Fums, from mesophilic microorganisms, are utilized for industrial l-malate production. However, the low thermostability of these Fums is a limitation in industrial l-malate production. Therefore, an alternative Class ІІ Fum that shows high activity and thermostability is required to overcome this drawback. Thermophilic microalgae and cyanobacteria can use carbon dioxide as a carbon source and are easy to cultivate. Among them, *Cyanidioschyzon merolae* and *Thermosynechococcus elongatus* are model organisms to study cell biology and structural biology, respectively. We biochemically analyzed Class ІІ Fums from *C. merolae* (*Cm*FUM) and *T. elongatus* (*Te*Fum). Both *Cm*FUM and *Te*Fum preferentially catalyzed fumarate hydration. The catalytic activity of *Cm*FUM for fumarate hydration in the optimum conditions (52°C and pH 7.5) is higher compared to those of Class ІІ Fums from other organisms and *Te*Fum. Thermostability tests of *Cm*FUM revealed that *Cm*FUM showed higher thermostability than those of Class ІІ Fums from other microorganisms. The yield of l-malate obtained from fumarate hydration catalyzed by *Cm*FUM was 75–81%. In summary, *Cm*Fum has suitable properties for efficient l-malate production.

## Introduction

Fumarase, or fumarate hydratase (EC 4.2.1.2; hereafter referred to as Fum) is one of the enzymes of the tricarboxylic acid (TCA) cycle and catalyzes the reversible hydration/dehydration reaction of fumarate to l-malate. Based on biochemical analyses of isozymes of Fum from *Escherichia coli*, these enzymes are divided into two biochemically distinct classes named Class І and ІІ (Woods et al., 1988). Amino acid sequence analysis revealed that there is no overall homology between Class І and ІІ Fums (Woods et al., 1988). There is an approximately 40% difference between the amino acid sequences of the Class ІІ Fums from eukaryotes and prokaryotes (Sacchettini et al., 1988). Class І Fums are thermolabile homo-dimeric enzymes, whereas Class ІІ Fums are thermostable homo-tetrameric enzymes (Woods et al., 1988).

l-Malate is used in various industrial applications such as acidulants, flavor enhancers, color fixatives, medicines, and antimicrobial agents (Liu et al., 2017). Fum has been used as a biocatalyst for industrial l-malate production (Liu et al., 2017). Class II Fum from the mesophilic bacterium *Corynebacterium glutamicum* (*Brevibacterium flavum*) can be used for l-malate production. l-Malate production using the *C. glutamicum* Fum requires heat treatment at 40–60°C for 10–300 min to repress by-product succinate formation because it is hard to separate succinate from l-malate (Terasawa et al., 1990). Thus, Fums used for l-malate production must maintain high activity after heat treatment. However, the stability of Class ІІ Fums from mesophilic microorganisms, including *C. glutamicum*, is insufficient at these temperatures (Takata et al., 1983; Keruchenko et al., 1992; Lin et al., 2007, 2018; Song et al., 2011). Class ІІ Fum from the highly thermophilic bacterium *Thermus thermophilus* shows high thermostability, with an optimum temperature of 85°C (Mizobata et al., 1998). However, this enzyme is not economically viable for l-malate production. The first reason is that maintaining the reaction temperature at 85°C is energy intensive. Second, the activity of this enzyme is lower than those of other Class ІІ Fums from mesophilic bacteria such as *C. glutamicum* (Lin et al., 2018). Thus, natural Class ІІ Fums from culturable microorganisms, which have suitable enzymatic properties for l-malate production, have not yet been identified. Previously, a Class ІІ Fum from *C. glutamicum* was modified by introducing three mutations to enhance thermostability (Lin et al., 2018).

Eukaryotic microalgae and cyanobacteria are microorganisms that perform oxygenic photosynthesis and can use carbon dioxide as the sole carbon source. In recent years, biotechnological applications of thermophilic microalgae and cyanobacteria have been gaining attention because these organisms do not compete for food sources and their growth at high temperatures prevents contamination with other microorganisms (Patel et al., 2019). The entire genomic sequences of the hot-spring red alga *Cyanidioschyzon merolae* and the hot-spring cyanobacterium, *Thermosynechococcus elongatus* are known (Ohta et al., 1998, 2003; Nakamura et al., 2002; Matsuzaki et al., 2004; Nozaki et al., 2007) and they can be easily cultivated. *C. merolae* is a eukaryote that has the simplest cellular structure and has been primarily used for cell biological studies so far (Kuroiwa et al., 1998). *T. elongatus* is the simplest photosynthetic organism that displays thermostability and therefore has been used for structural analyses of the photosynthetic system (Murray et al., 2007; Laughlin et al., 2019; Schuller et al., 2019; Zhang et al., 2019). However, biochemical analyses of their enzymes of primary metabolic pathways, such as the TCA cycle, have not yet been performed. Additionally, Fums from thermophilic microalgae and cyanobacteria, have not been biochemically characterized. Genome sequencing revealed that both *C. merolae* and *T. elongatus* have a sole Class ІІ Fum as a fumarase (Nakamura et al., 2002; Matsuzaki et al., 2004).

In this study, we biochemically characterized Fums from *C. merolae* (*Cm*FUM) and *T. elongatus* (*Te*Fum) and examined whether these Fums have suitable enzymatic properties for l-malate production.

## Materials and Methods

### Preparation of Expression Constructs of *Cm*FUM and *Te*Fum

The genomic regions containing *CmFUM* (CMD058C) and *TeFum* (tll1534) with N-terminal *BamHI* and C-terminal *XhoI* sites were commercially synthesized by Eurofin Genomics Japan (Tokyo, Japan). Codon usage was optimized for *E. coli*. The synthesized DNA fragments were cloned into the *BamHI-XhoI* site of the pGEX6P-1 vector (GE Healthcare, Little Chalfont, United Kingdom).

### Purification of *Cm*FUM and *Te*Fum

Glutathione-*S*-transferase (GST)-tagged *Cm*FUM and *Te*Fum were purified using affinity chromatography as described with a few alterations (Takeya et al., 2017). The *Cm*FUM and *Te*Fum constructs were transformed individually into *E. coli* BL21 (DE3) competent cells (BioDynamics Laboratory Inc., Tokyo, Japan). BL21 (DE3) cells were cultivated overnight in 1.5 L LB media at 30°C with shaking (150 rpm). During the cultivation of BL21 (DE3) cells, the expression of GST-tagged Fums was induced by 0.01 mM isopropyl β-D-1-thiogalactopyranoside (Wako Chemicals, Osaka, Japan). The cells were collected by repeated centrifugation (5,800 *g*, 2 min, 25°C) and transferred to 50 ml tubes containing PBS-T (1.37 M NaCl, 27 mM KCl, 81 mM Na_2_HPO_4_·12H_2_O, 14.7 mM KH_2_PO_4_, and 0.05% Tween-20). To dissolve the GST-tagged Fums in PBS-T, the cells were sonicated for 200 s at 20% intensity (model VC-750, EYELA, Tokyo, Japan). After centrifugation (14,200 *g*, 15 min, 4°C), 800 μl of glutathione-Sepharose 4B resin (GE Healthcare Japan, Tokyo, Japan) was added to the supernatant containing the GST-tagged Fums. To bind the GST-tagged Fums to glutathione-Sepharose 4B resin, the mixture was shaken for 1 h on ice. After centrifugation (5,800 *g*, 2 min, 4°C) to remove the supernatant, the resin was washed three and five times using 3 ml and 700 μl of PBS-T, respectively, to remove non-specific proteins. Thereafter, the GST-tagged Fums were eluted five times using 500 μl of GST elution buffer (50 mM Tris-HCl (pH 8.0) and 10 mM reduced glutathione) and concentrated using a VivaSpin 500 MWCO 50,000 device (Sartorius, Göttingen, Germany). The protein concentration was calculated using a Pierce BCA Protein Assay Kit (Thermo Scientific, Rockford, IL).

### Enzyme Assays for *Cm*FUM and *Te*Fum

The 1 ml assay solution of *Cm*FUM contains 100 mM Tris-HCl [pH 7.5 (fumarate hydration) or 8.5 (l-malate dehydration)], 5 pmol *Cm*FUM, and various concentrations of fumarate or l-malate. The 1 ml assay solution of *Te*Fum contains 100 mM Tris-HCl [pH 7.0 (fumarate hydration) or 7.5 (l-malate dehydration)], 30 pmol *Te*Fum, and various concentrations of fumarate or l-malate. The assay solution of *Cm*FUM and *Te*Fum before adding substrates was incubated for 5 min at 52°C and 50°C, respectively. Thereafter, various concentrations of fumarate or l-malate were added to the assay solution to start the reaction. The activities of *Cm*FUM and *Te*Fum were calculated by monitoring the changes in absorbance at *A*_240_ using a Shimadzu UV-1850 (Shimadzu, Kyoto, Japan). One unit of Fum activity was defined as the amount of Fum that converts 1 μmol fumarate or l-malate per min. The maximum reaction velocity (*V*_max_) and *K*_m_ (substrate concentration at 50% *V*_max_) of *Cm*FUM and *Te*Fum were calculated by curve fitting of the Michaelis-Menten equation using the Kaleida Graph ver. 4.5. The *k*_cat_ (turnover number) of *Cm*FUM and *Te*Fum was calculated from their *V*_max_ values.

### Thermostability Measurements of *Cm*FUM

Thermostability measurements of *Cm*FUM were performed as described previously (Lin et al., 2018). The 1 ml assay solution of *Cm*FUM contains 100 mM Tris-HCl (pH 7.5), 5 pmol *Cm*FUM, and 0.5 mM fumarate. To measure the *T*_50_^15^ (temperature where the activity becomes 50% after heat treatment for 15 min), the assay solution before adding fumarate was pre-incubated at each temperature for 15 min. Thereafter, the enzyme assay described in the previous section was performed. To measure *t*_1/2_ (time where the activity becomes 50% after heat treatment), the assay solution before adding fumarate was preincubated at 50°C for each time-point and then immediately cooled on ice for 1 min. Thereafter, the enzyme assay described in the previous section was performed.

### Analysis of the Reaction Catalyzed by *Cm*Fum When Using 200 mM Fumarate as a Substrate

The 500 μl assay solution contains 100 mM Tris-HCl (pH 7.5), 500 pmol *Cm*FUM, and 200 mM disodium fumarate. The assay solution before adding fumarate was pre-incubated for 5 min at 52°C. Thereafter, fumarate which was also pre-incubated for 5 min at 52°C was added to the assay solution and the reaction was started at 52°C. After the reaction for 5, 10, 20, 30, 40, 50, and 60 min, 50 μl of the assay solution was collected and the reaction was stopped by 100 mM HCl. The samples were analyzed by high-performance liquid chromatography (HPLC) using an LC-2000Plus System (JASCO, Tokyo, Japan). Organic acids were quantified using 0.2 mM bromothymol blue in 15 mM sodium phosphate buffer; peaks were detected at 445 nm, as described previously (Osanai et al., 2015).

### Analysis of the Reaction Catalyzed by *Cm*Fum When Using 1 M Fumarate as a Substrate

The 100 μl assay solution containing 100 mM Tris-HCl (pH 7.5), 50 pmol *Cm*FUM, and 1 M disodium fumarate was incubated at 52°C for 24 h. Thereafter, HCl was added to the assay solution to be 100 mM. The sample is analyzed by HPLC using an LC-2000Plus System (JASCO, Tokyo, Japan). Organic acids were quantified using 0.2 mM bromothymol blue in 15 mM sodium phosphate buffer and peaks were detected at 445 nm, as described previously (Osanai et al., 2015).

## Results

### Biochemical Properties of *Cm*FUM and *Te*Fum

**Figure 1:**
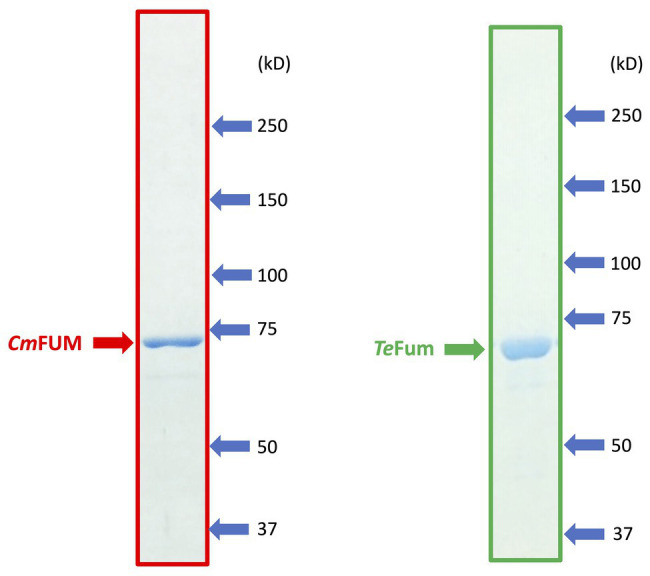
SDS-PAGE results after purification of GST-tagged *Cm*FUM (left) and *Te*Fum (right). SDS-PAGE gels (8%) were stained with InstantBlue (Expedion Protein Solutions, San Diego, CA).

**Figure 2:**
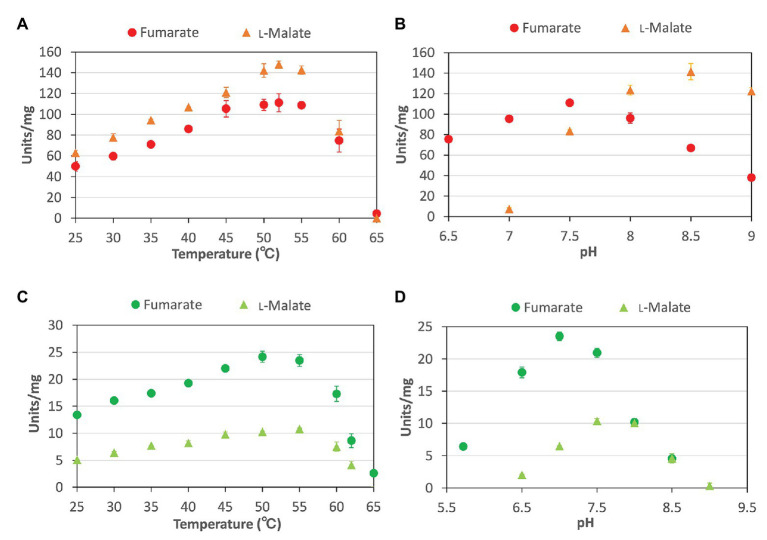
Temperature and pH dependence of *Cm*FUM and *Te*Fum **(A)** *Cm*FUM activity at each temperature. The measurements using fumarate and l-malate as substrates were performed at pH 7.5 and 8.5, respectively. The concentrations of fumarate and l-malate were 0.5 and 5 mM, respectively. **(B)** *Cm*FUM activity at each pH level. The measurements were performed at 52°C. The concentrations of fumarate and l-malate were 0.5 and 5 mM, respectively. **(C)** *Te*Fum activity at each temperature. The measurements using fumarate and l-malate as substrates were performed at pH 7.0 and 7.5, respectively. The concentrations of fumarate and l-malate were 0.5 and 1 mM, respectively. **(D)** *Te*Fum activity at each pH level. The measurements were performed at 50°C. The concentrations of fumarate and l-malate were 0.5 and 1 mM, respectively. The circles and triangles in Figure 2 indicate the activity using fumarate and l-malate as substrates, respectively. All data in Figure 2 indicate the mean ± SD obtained from three independent experiments.

To characterize the biochemical properties of *Cm*FUM and *Te*Fum, we purified *Cm*FUM and *Te*Fum as GST-tagged proteins using affinity chromatography (Figure 1). We first measured their activities using fumarate as a substrate (hereafter “the activity for fumarate”) and l-malate as a substrate (hereafter “the activity for l-malate”) at different temperatures and pH values (Figure 2). *Cm*FUM showed the highest activity for both substrates at 52°C (Figure 2A). *Cm*FUM showed the highest activity for fumarate and l-malate at pH 7.5 and 8.5, respectively, (Figure 2B). We then set the measurement conditions of *Cm*FUM activities for fumarate at 52°C and pH 7.5 and for l-malate at 52°C and pH 8.5. *Te*Fum consistently showed high activity for both substrates at 45–55°C (Figure 2C) and showed the highest activity for fumarate and l-malate at pH 7.0 and 7.5, respectively (Figure 2D). We then set the measurement conditions of *Te*Fum activities for fumarate at 50°C and pH 7.0, and for l-malate at 50°C and pH 7.5.

**Figure 3:**
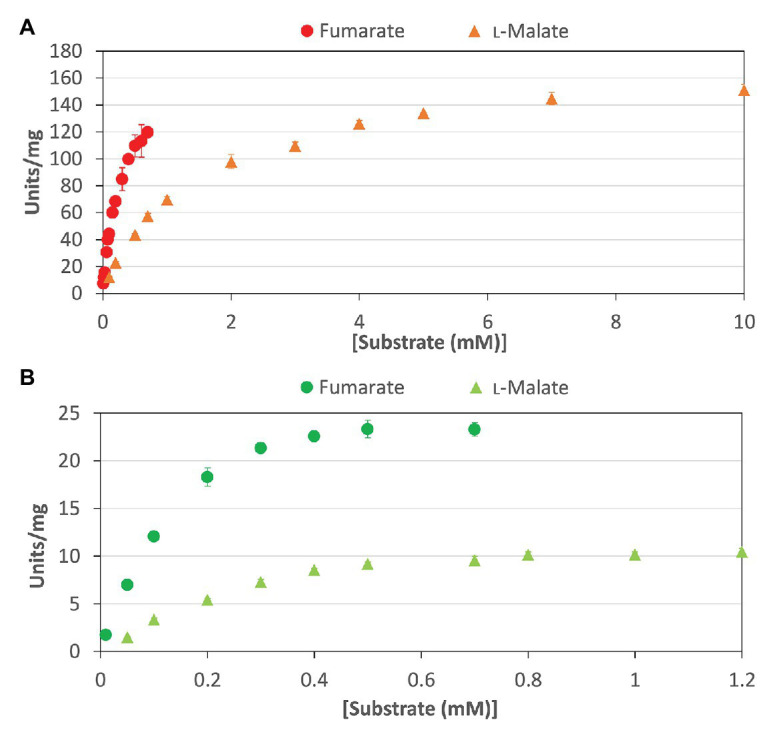
Saturation curves of *Cm*FUM and *Te*Fum for substrates. **(A)** Saturation curves of *Cm*FUM for fumarate (circles) and l-malate (triangles). The measurements using fumarate and l-malate as substrates were performed at 52°C and pH 7.5, and 52°C and pH 8.5, respectively. **(B)** Saturation curves of *Te*Fum for fumarate (circles) and l-malate (triangles). The measurements using fumarate and l-malate as substrates were performed at 50°C and pH 7.0, and 50°C and pH 7.5, respectively. All the data in Figure 3 indicate the mean ± SD obtained from three independent experiments.

To calculate the kinetic parameters of *Cm*FUM and *Te*Fum, the enzymatic activities were measured at different substrate concentrations (Figure 3). The saturation curves of *Cm*FUM and *Te*Fum for both substrates were not sigmoidal but hyperbolic (Figure 3) and the kinetic parameters of *Cm*FUM and *Te*Fum were calculated using the Michaelis-Menten equation (Table 1). The *K*_m_ (substrate concentration at 50% *V*_max_) and *k*_cat_ (turnover number) of *Cm*FUM for fumarate were 0.27 ± 0.05 mM and 235 ± 22 s^−1^, respectively (Table 1). The *K*_m_ and *k*_cat_ of *Cm*FUM for l-malate were 1.49 ± 0.12 mM and 244 ± 6 s^−1^, respectively (Table 1). The *k*_cat_/*K*_m_ (catalytic efficiency) of *Cm*FUM for fumarate (872 ± 68 s^−1^ mM^−1^) was 5.3-fold higher than that for l-malate (164 ± 9 s^−1^ mM^−1^; Table 1). The *K*_m_ and *k*_cat_ of *Te*Fum for fumarate were 0.14 ± 0.02 mM and 37 ± 2 s^−1^, respectively (Table 1). The *K*_m_ and *k*_cat_ of *Te*Fum for l-malate were 0.20 ± 0.01 mM and 15 ± 0.3 s^−1^, respectively, (Table 1). The *k*_cat_/*K*_m_ of *Te*Fum for fumarate (278 ± 23 s^−1^ mM^−1^) was 3.7-fold higher than that for l-malate (76 ± 1 s^−1^ mM^−1^).

**Table 1:** Kinetic parameters of *Cm*FUM and *Te*Fum.

Enzyme	Substrate	*K*_m_ (mM)	*k*_cat_ (s^−1^)	*k*_cat_/*K*_m_ (s^−1^ mM^−1^)	Ratio *k*_cat_/*K*_m_ (fumarate/l-malate)
*Cm*FUM	Fumarate	0.27 ± 0.05	235 ± 22	872 ± 68	5.3
	l-Malate	1.49 ± 0.12	244 ± 6	164 ± 9	
*Te*Fum	Fumarate	0.14 ± 0.02	37 ± 2	278 ± 23	3.7
	l-Malate	0.20 ± 0.01	15 ± 0.3	76 ± 1	

The measurement conditions are described in the legend of Figure 3. Data represent the mean ± SD obtained from three independent data points.

**Figure 4:**
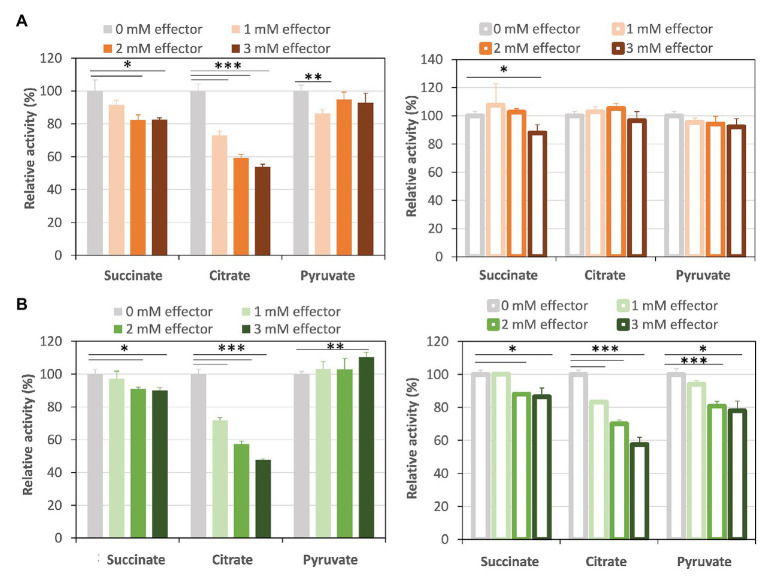
Effects of three organic acids on *Cm*FUM and *Te*Fum activity. **(A)** *Cm*FUM activity using fumarate (left) and l-malate (right) as a substrate in the presence of organic acids. The measurements using fumarate and l-malate as substrates were performed at 52°C and pH 7.5, and 52°C and pH 8.5, respectively. The concentrations of fumarate and l-malate were the *K*_m_ values of *Cm*FUM, 0.27 mM and 1.49 mM, respectively. **(B)** *Te*Fum activity using fumarate (left) and l-malate (right) as a substrate in the presence of organic acids. The measurements using fumarate and l-malate as substrates were performed at 50°C and pH 7.0, and 50°C and pH 7.5, respectively. The concentrations of fumarate and l-malate were the *K*_m_ values of *Te*Fum, 0.14 mM and 0.20 mM, respectively. All organic acids used as effectors were sodium salts. All the enzymatic activities in Figure 4 are represented by relative activities and the activity in the absence of effectors (gray bar) was set at 100%. All data in Figure 4 indicate the mean ± SD obtained from three independent experiments. All asterisks in Figure 4 indicate statistically significant differences between the absence and presence of the effector (Welch’s *t-*test; ^*^p < 0.05, ^**^p < 0.01, ^***^p < 0.005). All *p*-values obtained from Welch’s *t-*test in (A,B) are listed in Supplementary Tables S1 and S2, respectively.

Succinite, citrate, and pyruvate act as effectors of Class ІІ Fums from higher plant *Arabidopsis thaliana* (mitochondrial Fum; Zubimendi et al., 2018) and mesophilic cyanobacterium *Synechocystis* sp. PCC 6803 (hereafter *Synechocystis* 6803; Katayama et al., 2019). We examined the effects of the three organic acids on *Cm*FUM and *Te*Fum activities (Figure 4). The three organic acids decreased the *Cm*FUM activity for fumarate (Figure 4A). Succinate decreased the *Cm*FUM activity for l-malate (Figure 4A). Succinate and citrate decreased the *Te*Fum activity for fumarate (Figure 4B). In contrast, pyruvate increased the *Te*Fum activity for fumarate (Figure 4B). The three organic acids decreased the *Te*Fum activity for l-malate (Figure 4B).

### Further Biochemical Analyses of *Cm*FUM for L-Malate Production

**Figure 5:**
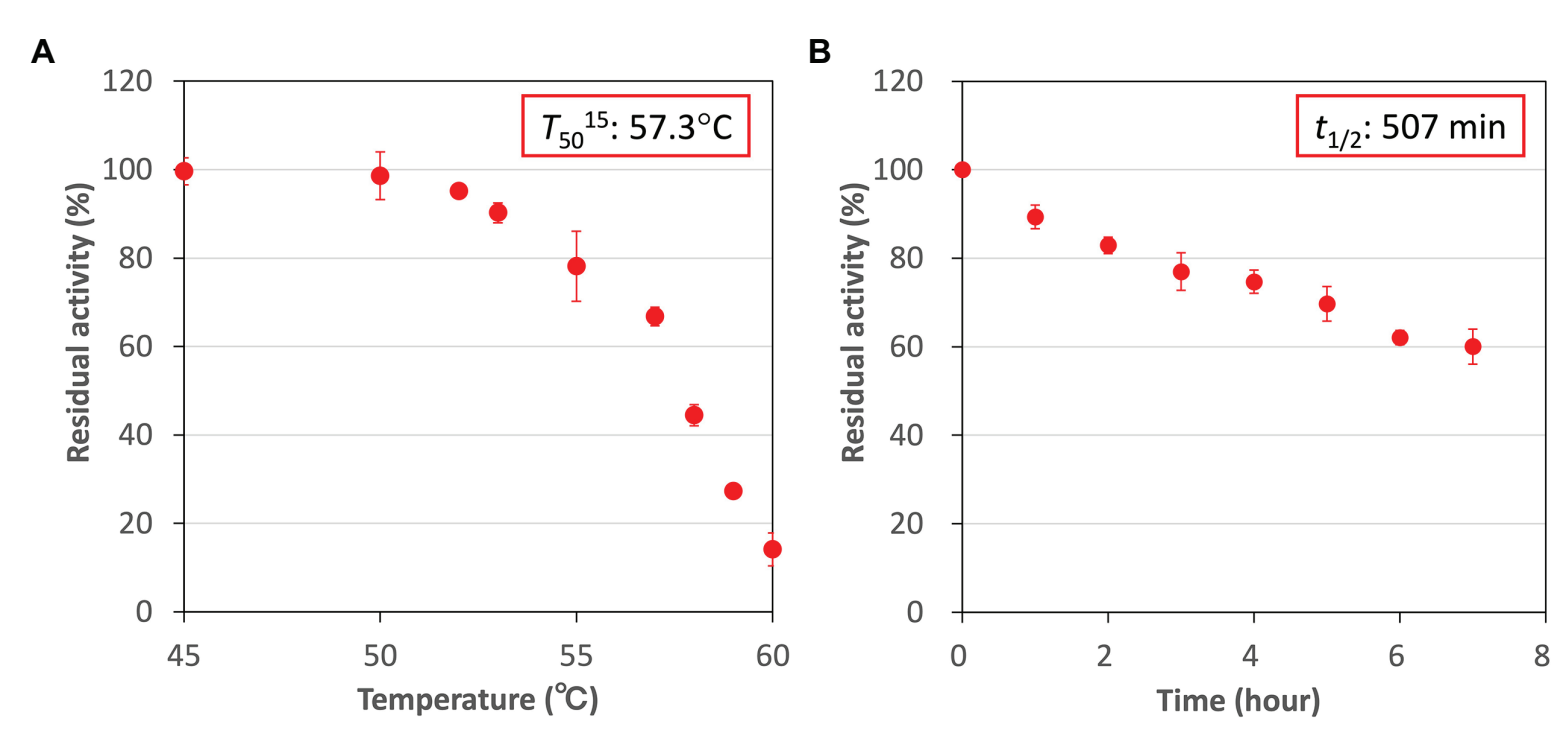
Thermostability of *Cm*FUM. **(A)** *Cm*FUM activity after heat treatment at each temperature for 15 min. The measurements were performed at pH 7.5. The concentration of fumarate was 0.5 mM. The *T*_50_^15^ was calculated using a linear equation obtained from six values (53–60°C). **(B)** *Cm*FUM activity after heat treatment at 50°C for each time-point. The measurements were performed at pH 7.5. The concentration of fumarate was 0.5 mM. The *t*_1/2_ was calculated using a linear equation obtained from all the values. All the enzymatic activities in Figure 5 are represented by residual activities, and the activity without heat-treatment was set at 100%. All the data in Figure 5 show the mean ± SD obtained from three independent experiments.

Higher activity and specificity for fumarate were seen in *Cm*FUM than in *Te*Fum (Table 1). Therefore, we examined the important enzymatic property for l-malate production, thermostability of *Cm*FUM (Figure 5). The residual activity of *Cm*FUM for fumarate after heat treatment for 15 min decreased linearly depending on the heat treatment temperature within the range of 53–60°C (Figure 5A). The *T*_50_^15^ (temperature where the activity becomes 50% after heat treatment for 15 min) was calculated as 57.3°C using a linear equation (Figure 5A). In addition, the residual activity of *Cm*FUM for fumarate after heat treatment at 50°C decreased linearly depending on the length of heat treatment (Figure 5B). The *t*_1/2_ (time where the activity becomes 50% after heat treatment) at 50°C was calculated as 507 min using a linear equation (Figure 5B).

**Figure 6:**
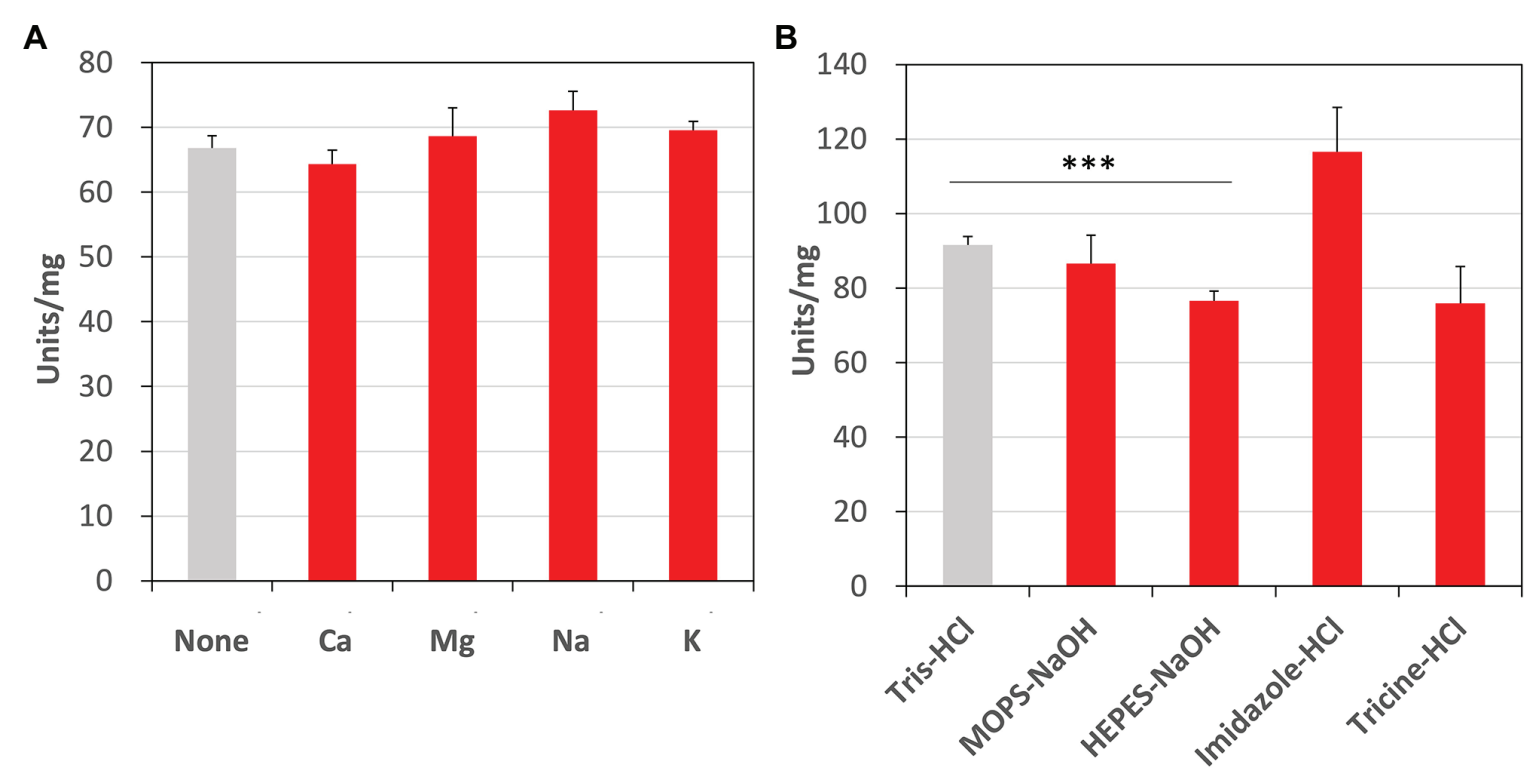
Effects of metal cations and buffer solutions on *Cm*FUM activity. **(A)** *Cm*FUM activity in the presence of 5 mM metal cations. The measurement was performed at 52°C and pH 7.5. The concentration of fumarate was the *K*_m_ of *Cm*FUM, 0.27 mM. Ca: CaCl_2_, Mg: MgCl_2_, Na: NaCl, K: KCl **(B)** *Cm*FUM activity in 100 mM buffer solutions. The measurement was performed at 52°C and pH 7.5. The concentration of fumarate was 0.5 mM. The asterisk indicates a statistically significant difference between the activity in Tris-HCl and HEPES-NaOH buffer (Welch’s *t*-test; ^***^p < 0.005). All *p*-values obtained from Welch’s *t-*test in (A,B) are listed in Supplementary Tables S3 and S4, respectively.

Additionally, we examined the effects of metal cations and buffer solutions on *Cm*FUM activity for fumarate and determined the condition where *Cm*FUM shows the highest activity for fumarate (Figure 6). *Cm*FUM activity for fumarate did not change in the presence of monovalent and divalent metal cations (Figure 6A). *Cm*FUM activity for fumarate in HEPES-NaOH buffer was slightly lower than that in Tris-HCl buffer (Figure 6B). *Cm*FUM activity for fumarate in each of the other three buffers tested was not significantly different from that in Tris-HCl buffer (Figure 6B).

**Figure 7:**
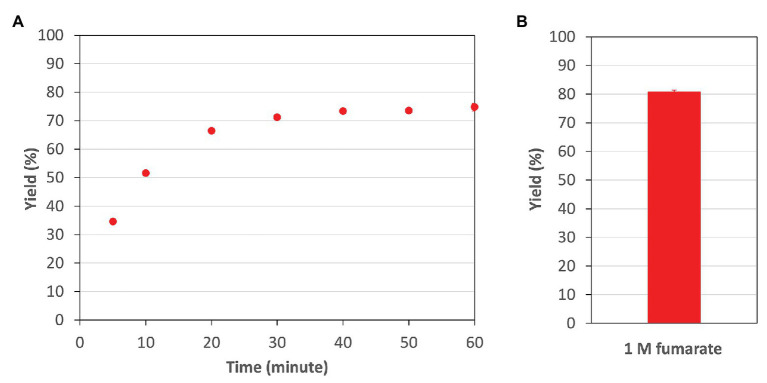
The yield of l-malate obtained from fumarate hydration catalyzed by *Cm*FUM. **(A)** The yield of l-malate when using 200 mM fumarate as a substrate. The concentration of *Cm*Fum was 1 μM. The measurement was performed at 52°C and pH 7.5. **(B)** The yield of l-malate when using 1 M fumarate as a substrate. The concentration of *Cm*Fum was 0.5 μM. The measurement was performed at 52°C and pH 7.5 for 24 h. All the data in Figure 7 show the mean ± SD obtained from three independent experiments.

Finally, in the optimum conditions of *Cm*FUM, we analyzed the reaction catalyzed by *Cm*FUM using high concentrations (industrial level) of fumarate as a substrate. *Cm*FUM showed enzymatic activity in the presence of 200 mM and 1 M fumarate (Figure 7). When using 200 mM fumarate as a substrate of *Cm*FUM, the yield of l-malate increased depending on reaction time, and the yield in an equilibrium state was 75% (Figure 7A). Also, the yield of l-malate when using 1 M fumarate as a substrate of *Cm*FUM was 81% (Figure 7B). An unwanted by-product in l-malate production, succinate was not detected in all samples using high concentrations of fumarate as a substrate.

## Discussion

For the first time, Fums from a thermophilic microalga and cyanobacterium were purified and biochemically characterized. The optimum temperatures for the enzymatic activity of *Cm*FUM (52°C) and *Te*Fum (45–55°C) were higher than those of Class ІІ Fums from mesophilic microorganisms (30–45°C, four species: *Streptomyces coelicolor*, *Rhizopus oryzae*, *Synechocystis* 6803, and *Streptomyces lividans*; Lin et al., 2007; Song et al., 2011; Su et al., 2014; Katayama et al., 2019), similar to that of Class ІІ Fum from *Streptomyces thermovulgaris* (50°C; Lin et al., 2007), and lower than those of Class ІІ Fums from *T. thermophilus* (85°C; Mizobata et al., 1998) and the thermophilic archaebacterium *Sulfolobus solfataricus* (85°C; Puchegger et al., 1990; Figures 2A,C). *C. merolae* grows optimally at 46°C (Moriyama et al., 2008), suggesting that *Cm*FUM stably shows high activity at the optimum growth temperature. *T. elongatus* rapidly grows in the range of 50–60°C (Yamaoka et al., 1978). In *T. elongatus*, the activities of photosynthesis and entire electron transport were dependent on temperature and high in the range of 50–60°C (Yamaoka et al., 1978). It is suggested that *Te*Fum also becomes active at these growth temperatures. The optimum pH for *Cm*FUM (pH 7.5 for fumarate hydration; pH 8.5 for l-malate dehydration) and *Te*Fum (pH 7.0 for fumarate hydration; pH 7.5 for l-malate dehydration) were approximately the same as those of Class ІІ Fums from other organisms (pH 6.5–8.5, seven species: *R. oryzae*, *Synechocystis* 6803, *Saccharomyces cerevisiae*, *S. solfataricus*, *C. glutamicum*, *A. thaliana*, and *Homo sapience*; Puchegger et al., 1990; Keruchenko et al., 1992; Genda et al., 2006; Song et al., 2011; Zubimendi et al., 2018; Ajalla Aleixo et al., 2019; Katayama et al., 2019; Figures 2B,D). Intercellular pH of *C. merolae* and cyanobacteria is maintained near neutral where *Cm*FUM and *Te*Fum show enzymatic activities (Coleman and Colman, 1981; Zenvirth et al., 1985; Mangan et al., 2016). Unlike Class ІІ Fums from *A. thaliana* (mitochondrial Fum; Zubimendi et al., 2018) and *Synechocystis* 6803 (Katayama et al., 2019), there was a significant difference between the optimum pH for fumarate hydration and l-malate dehydration in *Cm*FUM and *Te*Fum (particularly *Cm*FUM; Figures 2B,D). Therefore, we can regulate the equilibrium of the reaction catalyzed by *Cm*FUM by adjusting the pH. This property would be beneficial for l-malate production using fumarase.

**Figure 8:**
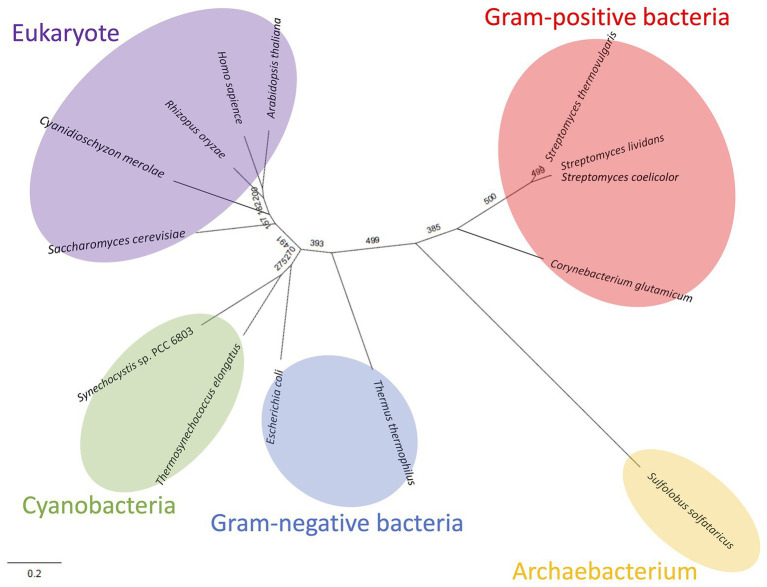
Phylogenetic analysis of biochemically characterized Class ІІ Fums. Sequence alignment of 14 Class ІІ Fums was performed using CLC Sequence Viewer ver. 8.0. A phylogenetic tree was generated by maximum likelihood method based on 423 conserved amino acid residues using PhyML online (http://www.atgc-montpellier.fr/phyml/). Bootstrap value obtained by 500 replications indicates the reliability of each branch.

**Figure 9:**
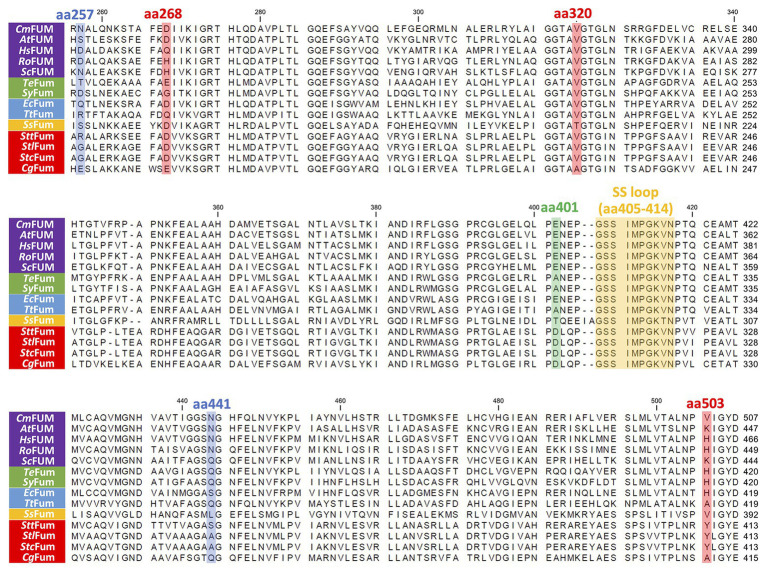
Amino acid sequence comparison of biochemically characterized Class ІІ Fums. Sequences of 14 Class ІІ Fums were obtained from GenBank and aligned using CLC Sequence Viewer ver. 8.0. The blue squares represent amino acid residues equivalent to position 257 and 441 of *Cm*FUM which contribute to the activity of Class ІІ Fum from *S. coelicolor* (Lin et al., 2007). The green square represents an amino acid residue equivalent to position 401 of *Cm*FUM which contributes to the activity of Class ІІ Fum from *Synechocystis* 6803 (Katayama et al., 2019). The red squares represent amino acid residues equivalent to position 268, 320, and 503 of *Cm*FUM which contribute to the thermostability of Class ІІ Fum from *C. glutamicum* (Lin et al., 2018). The yellow square represents a loop region containing the sequence GSSxxPxKxN (called a SS loop) which contributes to substrate binding and catalytic activity (Puthan Veetil et al., 2012). *At*FUM: Class ІІ Fum from *A. thaliana*, *Hs*FUM: Class ІІ Fum from *H. sapience*, *Ro*FUM: Class ІІ Fum from *R. oryzae*, *Sc*FUM: Class ІІ Fum from *S. cerevisiae*, *Sy*Fum: Class ІІ Fum from *Synechocystis* 6803, *Ec*Fum: Class ІІ Fum from *E. coli*, *Tt*Fum: Class ІІ Fum from *T. thermophilus*, *Ss*Fum: Class ІІ Fum from *S. solfataricus*, *Stt*Fum: Class ІІ Fum from *S. thermovulgaris*, *Stl*Fum: Class ІІ Fum from *S. lividans*, *Stc*Fum: Class ІІ Fum from *S. coelicolor*, *Cg*Fum: Class ІІ Fum from *C. glutamicum*.

Like Class ІІ Fums from other organisms (*A. thaliana*, *Synechocystis* 6803, *C. glutamicum*, and *H. sapience*; Genda et al., 2006; Zubimendi et al., 2018; Ajalla Aleixo et al., 2019; Katayama et al., 2019), *Cm*FUM and *Te*Fum preferentially catalyze fumarate hydration rather than l-malate dehydration (Table 1). The *K*_m_ of *Cm*FUM (0.27 mM) and *Te*Fum (0.14 mM) for fumarate were within the range of most Class ІІ enzymes (0.03–3.07 mM, nine species: *E. coli*, *C. glutamicum*, *R. oryzae*, *Synechocystis* 6803, *S. cerevisiae*, *S. solfataricus*, *A. thaliana*, *T. thermophilus*, and *H. sapience*; Woods et al., 1988; Puchegger et al., 1990; Keruchenko et al., 1992; Mizobata et al., 1998; Song et al., 2011; Lin et al., 2018; Zubimendi et al., 2018; Ajalla Aleixo et al., 2019; Katayama et al., 2019; Table 1). The *k*_cat_ of *Cm*FUM (235 s^−1^) and *Te*Fum (37 s^−1^) for fumarate were within the range of Class ІІ Fums (21–513 s^−1^, four species: *C. glutamicum*, *Synechocystis* 6803, *A. thaliana*, and *H. sapience*, Lin et al., 2018; Zubimendi et al., 2018; Ajalla Aleixo et al., 2019; Katayama et al., 2019; Table 1). The *k*_cat_/*K*_m_ of *Cm*FUM (872 s^−1^ mM^−1^) for fumarate was similar to that of Class ІІ Fum from *H. sapience* (850 s^−1^ mM^−1^; Ajalla Aleixo et al., 2019), and higher than those of cyanobacterial Class ІІ Fums (*Synechocystis* 6803: 415 s^−1^ mM^−1^, *Te*Fum: 278 s^−1^ mM^−1^; Katayama et al., 2019) and Class ІІ Fums from *C. glutamicum* (247 s^−1^ mM^−1^; Lin et al., 2018) and *A. thaliana* (30 s^−1^ mM^−1^; Zubimendi et al., 2018; Table 1). Thus, *Cm*FUM shows high catalytic activity for fumarate hydration. Phylogenetic analysis of biochemically characterized Class ІІ Fums revealed that the catalytic activities of closely related enzymes are not necessarily conserved (Figure 8). This suggests that some amino acid residues and motifs affect the activities of Class ІІ Fums. A SS loop, a motif contributing to substrate binding and catalytic activity (Puthan Veetil et al., 2012) was highly conserved in Class ІІ Fums (Figure 9). In contrast, a combination of a total of three amino acid residues that contribute to the activities of Class ІІ Fums from *S. coelicolor* (equivalent to position 257 and 441 of *Cm*FUM; Lin et al., 2007) and *Synechocystis* 6803 (equivalent to position 401 of *Cm*FUM; Katayama et al., 2019) was different for each Class ІІ Fum (Figure 9). This combination of the three amino acid residues might bring diversity to the catalytic activities of Class ІІ Fums and contribute to the high catalytic activity of *Cm*FUM. Unlike higher plants and algae, *C. merolae* do not possess an NAD^+^-dependent malic enzyme in mitochondria, so that the pyruvate transport to mitochondria is essential to perform aerobic respiration (Kuroiwa et al., 2017). The respiratory oxygen consumption of *C. merolae* drastically increases when not organic acids in the TCA cycle such as l-malate but pyruvate is added to the cells as an exogenous substrate (Moriyama et al., 2015). This suggests that the pyruvate generation in glycolysis is a rate-limiting step of the aerobic respiration and the TCA cycle in *C. merolae* actively works for energy production unlike that in cyanobacteria (Wan et al., 2017). The high catalytic activity of *Cm*FUM supports this hypothesis.

Similar to Class ІІ Fums from higher plants (Zubimendi et al., 2018) and *Synechocystis* 6803 (Katayama et al., 2019), both *Cm*FUM and *Te*Fum were inhibited by succinate and citrate (Figure 4), suggesting a common mechanism of inhibition of Class ІІ Fums from photosynthetic organisms. In contrast, Class ІІ Fum from *C. glutamicum* is not inhibited by succinate and citrate (Genda et al., 2006). Analyses of the effects of pyruvate on the activities for fumarate and l-malate revealed that pyruvate moves the equilibrium of the reaction catalyzed by *Cm*FUM and *Te*Fum to l-malate dehydration and fumarate hydration, respectively (Figure 4). Pyruvate also moves the equilibrium of the reaction catalyzed by Class ІІ Fums from *A. thaliana* (mitochondrial Fum) and *Synechocystis* 6803 to l-malate dehydration and fumarate hydration, respectively (Zubimendi et al., 2018; Katayama et al., 2019). Phylogenetic analysis of biochemically characterized Class ІІ Fums revealed that eukaryotic Class ІІ Fums form an independent cluster, not including cyanobacterial Class ІІ Fums (Figure 8). These suggest that pyruvate affects the equilibrium of the reaction catalyzed by Class ІІ Fums from photosynthetic organisms and the effects are different between Class ІІ Fums from photosynthetic eukaryotes and cyanobacteria. The difference in metabolism and physiological characteristics between photosynthetic eukaryotes and cyanobacteria might be associated with the effects of pyruvate on their Class ІІ Fums.

Additional biochemical analyses of *Cm*FUM clarified whether this enzyme has suitable enzymatic properties for efficient l-malate production. The *T*_50_^15^ of *Cm*FUM (57.3°C) was higher than both the Class ІІ Fum from *C. glutamicum* (44.8°C) as well as its thermostable mutant (54.6°C; Lin et al., 2018; Figure 5A). Moreover, the *t*_1/2_ at 50°C of the Class ІІ Fum from *C. glutamicum* is 1 min (Lin et al., 2018), and the Class ІІ Fums from *S. cerevisiae* and *S. coelicolor* are immediately denatured at 50°C (Keruchenko et al., 1992; Lin et al., 2007). Class ІІ Fum from *S. thermovulgaris* shows higher thermostability than these mesophilic Class ІІ Fums and its *t*_1/2_ at 50°C is 300 min (Lin et al., 2007). *Cm*Fum showed higher thermostability than the Class ІІ Fum from *S. thermovulgaris* and its *t*_1/2_ at 50°C of *Cm*FUM was 507 min (Figure 5B). These suggest that *Cm*FUM can show high activity after heat treatment in l-malate production. Phylogenetic analysis of biochemically characterized Class ІІ Fums revealed that as well as the catalytic activities, and the thermostability of closely related enzymes is not necessarily conserved (Figure 8). In the Class ІІ Fum from *C. glutamicum*, three amino acid residues equivalent to position 268, 320, and 503 of *Cm*FUM contribute to the thermostability (Lin et al., 2018; Figure 9). Amino acid substitution equivalent to position 320 and 503 of *Cm*FUM to valine enhances the thermostability of the Class ІІ Fum from *C. glutamicum* (Lin et al., 2018). This suggests that the valine at position 320 and 503 of *Cm*Fum contribute to the high thermostability of *Cm*FUM (Figure 9). In the Class ІІ Fum from *Synechocystis* 6803, the activity for fumarate decreased in the presence of Na^+^ (Katayama et al., 2019). However, *Cm*FUM activity for fumarate did not change in the presence of monovalent and divalent metal cations (Figure 6A). In industrial l-malate production using fumarase, fumarate salts exist as sodium and calcium salts, which are easy to dissolve in water and do not affect the pH of the reaction and thus, can be used as fumarase substrates (Terasawa et al., 1990). *Cm*FUM, which is insensitive to metal cations, can use these fumarate salts as substrates. *Cm*FUM consistently showed enzymatic activity in four buffer solutions except for the HEPES-NaOH buffer (Figure 6B). Considering the costs of the buffer solutions, we believe that Tris-HCl is a suitable buffer for *Cm*FUM. The yield of l-malate when using *Cm*FUM (75–81%) was higher than the yields when using Class ІІ Fums from *C. glutamicum* (Chibata et al., 1987) and *T. thermophilus* (Ninh et al., 2013; Both are 70%; Figure 7). The yield of l-malate when using Class ІІ Fum from *R. oryzae* is expected to be 75–80% (Naude and Nicol, 2018).

In this study, we characterized the biochemical properties of Class ІІ Fums from a thermophilic microalga and cyanobacterium. We demonstrated that *Cm*FUM has suitable enzymatic properties for efficient l-malate production such as high activity and thermostability. The optimizations of l-malate production using *Cm*FUM such as the utilization of a whole-cell biocatalyst and reactor will be future developments.

## Data Availability Statement

The raw data supporting the conclusions of this article will be made available by the authors, without undue reservation.

## Author Contributions

SI designed the study, analyzed the data, and wrote the manuscript. KI and HS performed the experiments and analyzed the data. TO designed the study and wrote the manuscript. All authors contributed to the article and approved the submitted version.

### Conflict of Interest

The authors declare that the research was conducted in the absence of any commercial or financial relationships that could be construed as a potential conflict of interest.

## References

[ref1] Ajalla Aleixo M. A. Rangel V. L. Rustiguel J. K. de Pádua R. A. P. Nonato M. C. (2019). Structural, biochemical and biophysical characterization of recombinant human fumarate hydratase. FEBS J. 286, 1925–1940. 10.1111/febs.14782, PMID: 30761759

[ref2] Chibata I. Tosa T. Yamamoto K. (1987). Production of l-malic acid by immobilized microbial cells. Methods Enzymol. 136, 455–463. 10.1016/S0076-6879(87)36043-4

[ref3] Coleman J. R. Colman B. (1981). Inorganic carbon accumulation and photosynthesis in a blue-green alga as a function of external pH. Plant Physiol. 67, 917–921. 10.1104/pp.67.5.917, PMID: 16661792 PMC425800

[ref4] Genda T. Watabe S. Ozaki H. (2006). Purification and characterization of fumarase from *Corynebacterium glutamicum*. Biosci. Biotechnol. Biochem. 70, 1102–1109. 10.1271/bbb.70.1102 16717409

[ref5] Katayama N. Takeya M. Osanai T. (2019). Biochemical characterisation of fumarase C from a unicellular cyanobacterium demonstrating its substrate affinity, altered by an amino acid substitution. Sci. Rep. 9:10629. 10.1038/s41598-019-47025-7 31337820 PMC6650407

[ref6] Keruchenko J. S. Keruchenko I. D. Gladilin K. L. Zaitsev V. N. Chirgadze N. Y. (1992). Purification, characterization and preliminary X-ray study of fumarase from *Saccharomyces cerevisiae*. Biochim. Biophys. Acta 1122, 85–92. 10.1016/0167-4838(92)90131-V, PMID: 1633200

[ref7] Kuroiwa T. Kuroiwa H. Sakai A. Takahashi H. Toda K. Itoh R. (1998). The division apparatus of plastids and mitochondria. Int. Rev. Cytol. 181, 1–41. 10.1016/S0074-7696(08)60415-5 9522454

[ref8] Kuroiwa T. Miyagishima S. Y. Matsunaga S. Sato N. Nozaki H. Tanaka K. K. (eds.) (2017). *Cyanidioschyzon merolae*: A new model eukaryote for cell and organelle biology. Heidelberg, Germany: Springer.

[ref9] Laughlin T. G. Bayne A. N. Trempe J. F. Savage D. F. Davies K. M. (2019). Structure of the complex I-like molecule NDH of oxygenic photosynthesis. Nature 566, 411–414. 10.1038/s41586-019-0921-0, PMID: 30742075

[ref10] Lin W. Chan M. Goh L. L. Sim T. S. (2007). Molecular basis for thermal properties of *Streptomyces thermovulgaris* fumarase C hinge at hydrophilic amino acids R163, E170 and S347. Appl. Microbiol. Biotechnol. 75, 329–335. 10.1007/s00253-006-0822-7, PMID: 17245573

[ref11] Lin L. Wang Y. Wu M. Zhu L. Yang L. Lin J. (2018). Enhancing the thermostability of fumarase C from *Corynebacterium glutamicum* via molecular modification. Enzym. Microb. Technol. 115, 45–51. 10.1016/j.enzmictec.2018.04.010, PMID: 29859602

[ref12] Liu J. Li J. Shin H. D. Du G. Chen J. Liu L. (2017). Biological production of l-malate: recent advances and future prospects. World J. Microbiol. Biotechnol. 34:6. 10.1007/s11274-017-2349-8, PMID: 29214355

[ref13] Mangan N. M. Flamholz A. Hood R. D. Milo R. Savage D. F. (2016). pH determines the energetic efficiency of the cyanobacterial CO_2_ concentrating mechanism. Proc. Natl. Acad. Sci. U. S. A. 113, E5354–E5362. 10.1073/pnas.1525145113, PMID: 27551079 PMC5018799

[ref14] Matsuzaki M. Misumi O. Shin-I T. Maruyama S. Takahara M. Miyagishima S. Y.. (2004). Genome sequence of the ultrasmall unicellular red alga *Cyanidioschyzon merolae* 10D. Nature 428, 653–657. 10.1038/nature02398, PMID: 15071595

[ref15] Mizobata T. Fujioka T. Yamasaki F. Hidaka M. Nagai J. Kawata Y. (1998). Purification and characterization of a thermostable class II fumarase from *Thermus thermophilus*. Arch. Biochem. Biophys. 355, 49–55. 10.1006/abbi.1998.0693, PMID: 9647666

[ref16] Moriyama T. Mori N. Sato N. (2015). Activation of oxidative carbon metabolism by nutritional enrichment by photosynthesis and exogenous organic compounds in the red alga *Cyanidioschyzon merolae*: evidence for heterotrophic growth. Springerplus 4:559. 10.1186/s40064-015-1365-0 26435905 PMC4586181

[ref17] Moriyama T. Terasawa K. Fujiwara M. Sato N. (2008). Purification and characterization of organellar DNA polymerases in the red alga *Cyanidioschyzon merolae*. FEBS J. 275, 2899–2918. 10.1111/j.1742-4658.2008.06426.x, PMID: 18430024

[ref18] Murray J. W. Maghlaoui K. Barber J. (2007). The structure of allophycocyanin from *Thermosynechococcus elongatus* at 3.5 Å resolution. Acta Crystallogr. Sect. F Struct. Biol. Commun. 63, 998–1002. 10.1107/S1744309107050920 PMC2344114 18084078

[ref19] Nakamura Y. Kaneko T. Sato S. Ikeuchi M. Katoh H. Sasamoto S.. (2002). Complete genome structure of the thermophilic cyanobacterium *Thermosynechococcus elongatus* BP-1. DNA Res. 9, 123–130. 10.1093/dnares/9.4.123, PMID: 12240834

[ref20] Naude A. Nicol W. (2018). Malic acid production through the whole-cell hydration of fumaric acid with immobilised *Rhizopus oryzae*. Biochem. Eng. J. 137, 152–161. 10.1016/j.bej.2018.05.022

[ref21] Ninh P. H. Honda K. Yokohigashi Y. Okano K. Omasa T. Ohtake H. (2013). Development of a continuous bioconversion system using a thermophilic whole-cell biocatalyst. Appl. Environ. Microbiol. 79, 1996–2001. 10.1128/AEM.03752-12, PMID: 23335777 PMC3592215

[ref22] Nozaki H. Takano H. Misumi O. Terasawa K. Matsuzaki M. Maruyama S. (2007). A 100%-complete sequence reveals unusually simple genomic features in the hot-spring red alga *Cyanidioschyzon merolae*. BMC Biol. 5:28. 10.1186/1741-7007-5-28 17623057 PMC1955436

[ref23] Ohta N. Matsuzaki M. Misumi O. Miyagishima S. Y. Nozaki H. Tanaka K.. (2003). Complete sequence and analysis of the plastid genome of the unicellular red alga *Cyanidioschyzon merolae*. DNA Res. 10, 67–77. 10.1093/dnares/10.2.67, PMID: 12755171

[ref24] Ohta N. Sato N. Kuroiwa T. (1998). Structure and organization of the mitochondrial genome of the unicellular red alga *Cyanidioschyzon merolae* deduced from the complete nucleotide sequence. Nucleic Acids Res. 26, 5190–5198. 10.1093/nar/26.22.5190, PMID: 9801318 PMC147977

[ref25] Osanai T. Shirai T. Iijima H. Nakaya Y. Okamoto M. Kondo A.. (2015). Genetic manipulation of a metabolic enzyme and a transcriptional regulator increasing succinate excretion from unicellular cyanobacterium. Front. Microbiol. 6:1064. 10.3389/fmicb.2015.01064, PMID: 26500619 PMC4594341

[ref26] Patel A. Matsakas L. Rova U. Christakopoulos P. (2019). A perspective on biotechnological applications of thermophilic microalgae and cyanobacteria. Bioresour. Technol. 278, 424–434. 10.1016/j.biortech.2019.01.063, PMID: 30685131

[ref27] Puchegger S. Redl B. Stöffler G. (1990). Purification and properties of a thermostable fumarate hydratase from the archaeobacterium *Sulfolobus solfataricus*. J. Gen. Microbiol. 136, 1537–1541. 10.1099/00221287-136-8-1537, PMID: 2124611

[ref28] Puthan Veetil V. Fibriansah G. Raj H. Thunnissen A. M. Poelarends G. J. (2012). Aspartase/fumarase superfamily: a common catalytic strategy involving general base-catalyzed formation of a highly stabilized aci-carboxylate intermediate. Biochemistry 51, 4237–4243. 10.1021/bi300430j, PMID: 22551392

[ref29] Sacchettini J. C. Frazier M. W. Chiara D. C. Banaszak L. J. Grant G. A. (1988). Amino acid sequence of porcine heart fumarase. Biochem. Biophys. Res. Commun. 153, 435–440. 10.1016/S0006-291X(88)81243-9, PMID: 3377794

[ref30] Schuller J. M. Birrell J. A. Tanaka H. Konuma T. Wulfhorst H. Cox N.. (2019). Structural adaptations of photosynthetic complex I enable ferredoxin-dependent electron transfer. Science 363, 257–260. 10.1126/science.aau3613, PMID: 30573545

[ref31] Song P. Li S. Ding Y. Xu Q. Huang H. (2011). Expression and characterization of fumarase (FUMR) from *Rhizopus oryzae*. Fungal Biol. 11, 49–53. 10.1016/j.funbio.2010.10.003 21215954

[ref32] Su R. R. Wang A. Hou S. T. Gao P. Zhu G. P. Wang W. (2014). Identification of a novel fumarase C from *Streptomyces lividans* TK54 as a good candidate for l-malate production. Mol. Biol. Rep. 41, 497–504. 10.1007/s11033-013-2885-8, PMID: 24307253

[ref33] Takata I. Tosa T. Chibata I. (1983). Stabilization of fumarase activity of *Brevibacterium flavum* cells by immobilization with k-carrageenan. Appl. Biochem. Biotechnol. 8, 31–38. 10.1007/BF02798346

[ref34] Takeya M. Hirai M. Y. Osanai T. (2017). Allosteric inhibition of phosphoenolpyruvate carboxylases is determined by a single amino acid residue in cyanobacteria. Sci. Rep. 7:41080. 10.1038/srep41080 28117365 PMC5259782

[ref35] Terasawa M Nara T Yukawa H Yamagata H Satoo Y (1990). *Method of preparing l-malic acid*. U.S. Patent No 4,912,043.

[ref36] Wan N. DeLorenzo D. M. He L. You L. Immethun C. M. Wang G.. (2017). Cyanobacterial carbon metabolism: fluxome plasticity and oxygen dependence. Biotechnol. Bioeng. 114, 1593–1602. 10.1002/bit.26287, PMID: 28295163

[ref37] Woods S. A. Schwartzbach S. D. Guest J. R. (1988). Two biochemically distinct classes of fumarase in *Escherichia coli*. Biochim. Biophys. Acta 954, 14–26. 10.1016/0167-4838(88)90050-7, PMID: 3282546

[ref38] Yamaoka T. Satoh K. Katoh S. (1978). Photosynthetic activities of a thermophilic blue-green alga. Plant Cell Physiol. 19, 943–954. 10.1093/oxfordjournals.pcp.a075684

[ref39] Zenvirth D. Volokita M. Kaplan A. (1985). Photosynthesis and inorganic carbon accumulation in the acidophilic alga *Cyanidioschyzon merolae*. Plant Physiol. 77, 237–239. 10.1104/pp.77.1.237, PMID: 16664017 PMC1064490

[ref40] Zhang S. Heyes D. J. Feng L. Sun W. Johannissen L. O. Liu H.. (2019). Structural basis for enzymatic photocatalysis in chlorophyll biosynthesis. Nature 574, 722–725. 10.1038/s41586-019-1685-2, PMID: 31645759

[ref41] Zubimendi J. P. Martinatto A. Valacco M. P. Moreno S. Andreo C. S. Drincovich M. F.. (2018). The complex allosteric and redox regulation of the fumarate hydratase and malate dehydratase reactions of *Arabidopsis thaliana* Fumarase 1 and 2 gives clues for understanding the massive accumulation of fumarate. FEBS J. 285, 2205–2224. 10.1111/febs.14483, PMID: 29688630

