# Supplementary material for: Fumarase From Cyanidioschyzon merolae Stably Shows High Catalytic Activity for Fumarate Hydration Under High Temperature Conditions
Source: Front Microbiol. 2020 Sep 16;11:2190. doi: 10.3389/fmicb.2020.560894 (PMC7525151; doi:10.3389/fmicb.2020.560894)
Supplement: Supplementary file 1 [file Data_Sheet_1.docx]

**Table S1.** List of *p*-values obtained from Welch’s *t*-test in figure 4A

| Effector | *p*-value |
| --- | --- |
| *Cm*FUM activity using fumarate as a substrate | |
| 1 mM succinate | 0.154 |
| 2 mM succinate | 0.028 |
| 3 mM succinate | 0.043 |
| 1 mM citrate | 0.001 |
| 2 mM citrate | 0.001 |
| 3 mM citrate | 0.001 |
| 1 mM pyruvate | 0.007 |
| 2 mM pyruvate | 0.195 |
| 3 mM pyruvate | 0.162 |
| *Cm*FUM activity using l-malate as a substrate | |
| 1 mM succinate | 0.485 |
| 2 mM succinate | 0.329 |
| 3 mM succinate | 0.048 |
| 1 mM citrate | 0.403 |
| 2 mM citrate | 0.135 |
| 3 mM citrate | 0.484 |
| 1 mM pyruvate | 0.144 |
| 2 mM pyruvate | 0.208 |
| 3 mM pyruvate | 0.131 |

**Table S2.** List of *p*-values obtained from Welch’s *t*-test in figure 4B

| Effector | *p*-value |
| --- | --- |
| *Te*FUM activity using fumarate as a substrate | |
| 1 mM succinate | 0.430 |
| 2 mM succinate | 0.024 |
| 3 mM succinate | 0.012 |
| 1 mM citrate | 0.001 |
| 2 mM citrate | 0.0001 |
| 3 mM citrate | 0.001 |
| 1 mM pyruvate | 0.371 |
| 2 mM pyruvate | 0.550 |
| 3 mM pyruvate | 0.009 |
| *Te*FUM activity using l-malate as a substrate | |
| 1 mM succinate | 0.872 |
| 2 mM succinate | 0.013 |
| 3 mM succinate | 0.029 |
| 1 mM citrate | 0.004 |
| 2 mM citrate | 0.0001 |
| 3 mM citrate | 0.0004 |
| 1 mM pyruvate | 0.079 |
| 2 mM pyruvate | 0.002 |
| 3 mM pyruvate | 0.010 |

**Table S3.** List of *p*-values obtained from Welch’s *t*-test in figure 6A

| Metal cations | *p*-value |
| --- | --- |
| Ca | 0.211 |
| Mg | 0.555 |
| Na | 0.054 |
| K | 0.125 |

**Table S4.** List of *p*-values obtained from Welch’s *t*-test in figure 6B

| Buffer solutions | *p*-value |
| --- | --- |
| MOPS-NaOH | 0.373 |
| HEPES-NaOH | 0.002 |
| Imidazole-HCl | 0.064 |
| Tricine-HCl | 0.103 |
